# Supplementary material for: Polyketide synthase gene clusters in the ericoid mycorrhizal fungus Oidiodendron maius: genome-wide prediction, expression profiling, and functional characterization of knockout mutants
Source: Front Plant Sci. 2026 Sep 16;17:1924430. doi: 10.3389/fpls.2026.1924430 (PMC13623903; doi:10.3389/fpls.2026.1924430)
Supplement: Supplementary file 1 [file SupplementaryFile1.zip › Supplementary Materials/Supplementary Folder 1.zip!/Oidma1_AssemblyScaffolds_Repeatmasked/knownclusterblast/cluster16/jgi.p_Oidma1_40823_mibig_hits.html]

| MIBiG Protein | Description | MIBiG Cluster | MiBiG Product | % ID | % Coverage | BLAST Score | E-value |
| --- | --- | --- | --- | --- | --- | --- | --- |
| EAA59569.1 | hypothetical\_protein | BGC0000057 | F9775\_biosynthetic\_gene\_cluster | 38 | 52.6198439242 | 265 | 1.7e-70 |
| CBF73514.1 | beta-glucosidase\_(Eurofung) | BGC0001121 | Orsellinic\_acid\_biosynthetic\_gene\_cluster | 38 | 52.6198439242 | 265 | 1.7e-70 |
| ADN68487.1 | SorL | BGC0000184 | Sorangicin\_biosynthetic\_gene\_cluster | 31 | 74.6934225195 | 230 | 7.8e-60 |
| AAS79445.1 | putative\_beta-glucosidase | BGC0000035 | Chalcomycin\_biosynthetic\_gene\_cluster | 32 | 45.3734671126 | 169 | 2.1e-41 |
| ACR50763.1 | periplasmic\_beta-glucosidase | BGC0000163 | Tetronasin\_biosynthetic\_gene\_cluster | 26 | 65.9977703456 | 142 | 2.1e-33 |
| AAM88355.1 | NbmF | BGC0000899 | Desosamine\_biosynthetic\_gene\_cluster | 33 | 20.8472686734 | 84 | 5.3e-16 |
| AAC68679.1 | beta-glucosidase | BGC0000898 | Desosamine\_biosynthetic\_gene\_cluster | 32 | 20.7357859532 | 83 | 1.2e-15 |
| ABB52530.1 | beta\_glucosidase | BGC0000047 | Dihydrochalcomycin\_biosynthetic\_gene\_cluster | 32 | 23.9687848384 | 83 | 1.5e-15 |
| AAU93797.1 | beta-glucosidase | BGC0000054 | Erythromycin\_biosynthetic\_gene\_cluster | 29 | 21.4046822742 | 72 | 2.1e-12 |
